# Schizonepeta tenuifolia Briq-Saposhnikovia divaricata decoction alleviates atopic dermatitis via downregulating macrophage TRPV1

**DOI:** 10.3389/fphar.2024.1413513

**Published:** 2024-08-27

**Authors:** Hongmin Li, Jinyu Liang, Peifeng Li, Xiangzheng Li, Qing Liu, Songxue Yang, Chunlei Zhang, Shun Liu, Yuan He, Cheng Tan

**Affiliations:** ^1^ Affiliated Hospital of Nanjing University of Chinese Medicine, Jiangsu Province Hospital of Chinese Medicine, Nanjing, China; ^2^ State Key Laboratory of Natural Medicines, School of Basic Medicine and Clinical Pharmacy, China Pharmaceutical University, Nanjing, China; ^3^ Key Laboratory of Medicinal Chemistry for Natural Resource, Ministry of Education, Yunnan Provincial Center for Research and Development of Natural Products, School of Chemical Science and Technology, Yunnan University, Kunming, China; ^4^ Jiangsu Provincial Key Laboratory for TCM Evaluation and Translational Development, School of Traditional Chinese Pharmacy, China Pharmaceutical University, Nanjing, Jiangsu, China

**Keywords:** atopic dermatitis, Schizonepeta tenuifolia -Saposhnikovia divaricata, TRPV1, macrophage, NF-κB

## Abstract

**Objective:**

Schizonepeta tenuifolia -Saposhnikovia divaricata (Jingjie-Fangfeng, JF) has been used for years to treat allergic inflammatory skin diseases like atopic dermatitis, but the specific effects and mechanisms of JF are still unclear.

**Purpose:**

We aim to investigate the therapeutic effect and mechanism of JF in MC903-induced atopic dermatitis-like model.

**Methods:**

JF decoction was subjected to rigorous HPLC and GC analysis. The JF decoction was then freshly prepared and administered to MC903-induced atopic dermatitis -like mice models to investigate its therapeutic effects. Our evaluation focused on several markers of inflammation including the TEWL index, ear thickness, swelling, and specific inflammation indicators such as TSLP, IL33, IgE, and immune cell presence at the lesion sites. We measured Transient Receptor Potential Vanilloid 1 (TRPV1) expression levels through immunofluorescent staining in skin tissue from both atopic dermatitis patients and the MC903-treated mice. Furthermore, TRPV1 expression and macrophage activation markers were measured in LPS/IFN-γ-stimulated Raw264.7 and THP-1 cell models *in vitro*. Additionally, we developed cell lines that overexpress TRPV1 and investigated how JF treatment affects NF-κB p65 phosphorylation in these cells to understand better the role of TRPV1 in atopic dermatitis.

**Results:**

The JF decoction met the standards outlined in the Chinese pharmacopeia. The JF decoction significantly alleviated inflammatory skin symptoms and helped restore skin barrier function. Additionally, it reduced the levels of IgE and pro-inflammatory cytokines TSLP, IL-33, and IL-4. There was also a noticeable decrease in mast cell infiltration and degranulation. Notably, JF decoction reduced infiltrated macrophages with limited affection on T cell infiltration. It also decreased F4/80^+^/TRPV1^+^ cells in atopic dermatitis mice and TRPV1 expression in LPS/IFNγ-stimulated microphages. Additionally, we observed that CD68^+^/TRPV1^+^ cells increased in human atopic dermatitis tissue. Further studies showed that JF water extract (JF-WE) suppressed TRPV1 expression in macrophages, potentially by affecting NF-κB p65 phosphorylation rather than the JAK-STAT6 pathway.

**Conclusion:**

This study offers initial evidence of the effectiveness of JF-WE in suppressing inflammation in atopic dermatitis. The therapeutic effect might stems from its ability to downregulate TRPV1 expression and subsequent NF-κB p65 phosphorylation in macrophages.

## Introduction

Atopic dermatitis is a common form of eczema characteristically marked by symptoms like redness, swelling, scaling, and dryness. Over 200 million individuals are affected globally and is especially prevalent among children (Langan et al., 2020; Ständer, 2021). Individuals with atopic dermatitis are at an increased risk for developing a range of other allergic conditions, collectively referred to as the “atopic March” (Li et al., 2022). This skin disorder is complex and heterogeneous, influenced by both immune system dysfunctions and genetic factors, making it difficult to manage uniformly across all patients.

Due to the complex and versatile characters of atopic dermatitis, it is unrealistic to expect uniform treatment responses across all patients. Various biological reagents and chemical drugs have been developed and implemented in clinical settings to address this complexity, including IL-4/IL-13-targeting antibody dupilumab and tralokinumab (Beck et al., 2014; Ratchataswan et al., 2021), IL-22-targeting antibody fezakinumab (Brunner et al., 2019) and JAK inhibitors baricitinib, abrocitinib and upadacitinib (Guttman-Yassky et al., 2019; Nakashima et al., 2022). Despite their therapeutic potential, it is important to note that even highly effective treatments like dupilumab have limitations. Although treatments like dupilumab have shown promise, they often have limitations, such as a lower efficacy in some patients and potential adverse effects (Silverberg et al., 2021), particularly with JAK inhibitors, which have been flagged with black box warnings.

Given the limitations of conventional treatments, there is growing interest in traditional medicine alternatives. The Traditional Chinese Medicine (TCM) formula Xiao-feng-San from classical ancient books of TCM, for example, has been widely used to manage allergic skin diseases like atopic dermatitis. This formula includes the herbs Schizonepeta tenuifolia Briq and Saposhnikovia divaricata, which have been identified as effective in treating atopic dermatitis (Cheng et al., 2011). Our previous studies suggested these herbs might work by influencing macrophage behavior and interacting with the ion channel TRPV1 (Shi et al., 2021), which plays a role in skin barrier function and inflammation.

Many molecular factors and essential effector cells have been reported to attribute to the pathogenesis of atopic dermatitis. As atopic dermatitis endotypes can be stratified into non-type 2-, type 2- and a mixed type-dominant, each involving distinct immunological pathways. For instance, the IL-22 pathway implicated in non-type 2 atopic dermatitis (Chan et al., 2018), while IgE and/or IL-4/IL-13 pathway (Dyjack et al., 2018) are prominent in type 2 atopic dermatitis and JAK/STAT pathway is relevant in mixed-type atopic dermatitis. In line with this, key effector cells include immune cells such as Th2 cells, innate lymphoid cells (ILCs), macrophages, and dendritic cells, as well as non-immune cells like keratinocytes and somatosensory neurons (Xiao et al., 2023). In its pathogenesis, macrophages, as key players of the innate immune system, contribute significantly to the pathogenesis of atopic dermatitis through their multifaceted roles. For instance, Macrophages have garnered attention due to their increased presence in the skin of atopic dermatitis patients compared to healthy donors (Reynolds et al., 2021; Rojahn et al., 2020). Meanwhile, dysregulation of macrophage function attributed to aberrant immune responses, perpetuating inflammation and tissue damage. Indeed, dysregulated macrophage activation leads to excessive production of pro-inflammatory cytokines, such as interleukin-1 (IL-1), interleukin-6 (IL-6), and tumor necrosis factor-alpha (TNF-α) (Kim et al., 2023; Kodali et al., 2013; Stepanenko et al., 2024; Tang et al., 2023). Signaling pathways such as the Toll-like receptor (TLR) pathway and the interleukin (IL)-23/IL-17 axis are implicated in macrophage activation and polarization, contributing to the pathogenesis of atopic dermatitis (Kim et al., 2018; Kwon et al., 2022; Schiller et al., 2006; Yamaguchi et al., 2023). However, in contrast to the pre-dominant IL-23/Th17 signaling in Psoriasis (PsO) (Yin et al., 2014), atopic dermatitis pathogenesis involves a complex interplay of various pathways and effector cells. Understanding the intricate interplay between macrophages and signaling pathways is crucial for developing targeted therapeutic interventions to alleviate the burden of atopic dermatitis.

Another intriguing group of triggering cells is the somatosensory neuron, which releases pro-inflammatory mediators that activate and sensitize transient receptor potential (TRP) channels on various cells, exacerbating symptoms of atopic dermatitis, such as cutaneous inflammation and itching (Lv et al., 2021; Meng et al., 2021). Transient Receptor Potential Vanilloid 1 (TRPV1) emerges as a crucial mediator in the pathogenesis of atopic dermatitis, a prevalent inflammatory skin disorder characterized by pruritus, erythema, and epidermal barrier dysfunction. TRPV1, a non-selective cation channel predominantly expressed in sensory neurons, plays a multifaceted role in atopic dermatitis through neurogenic inflammation, itch sensation, and immune modulation (Bao and Abraham, 2024; Dire et al., 1995; Xu et al., 2024). Activation of TRPV1 by various endogenous and exogenous stimuli triggers the release of pro-inflammatory neuropeptides, such as substance P and calcitonin gene-related peptide (CGRP), fostering neurogenic inflammation and exacerbating atopic dermatitis-associated itching (Lee et al., 2023). Emerging evidence suggests TRPV1 directly influences immune cells, particularly dendritic cells and mast cells, by modulating cytokine production and cell activation. TRPV1-mediated release of these pro-inflammatory cytokines, including TNF-α, IL-6, IL-1β, can further exacerbate inflammatory responses in the skin, contributing to the pathogenesis of inflammatory conditions like atopic dermatitis, leading to symptoms such as erythema, pruritus, and skin barrier dysfunction (Ahn et al., 2024; Kordulewska and Król-Grzymała, 2024). Moreover, dysregulated TRPV1 signaling contributes to the disruption of epidermal barrier integrity and sensory nerve sensitization, perpetuating the chronic inflammatory cascade characteristic of atopic dermatitis. Elucidating the intricate role of TRPV1 and its associated signaling pathways holds promise for identifying novel therapeutic targets and advancing the management of atopic dermatitis.

Here, we selected Schizonepeta tenuifolia Briq and Saposhnikovia divaricata from Xiao-Feng-San to evaluate their efficacy in a mouse model of atopic dermatitis induced by MC903. We focused on their potential to reduce skin inflammation, particularly through modulating macrophage polarization and enhancing skin barrier integrity. Our preliminary findings suggest that these herbs might regulate TRPV1 expression and influence associated inflammatory pathways, providing insights into their mechanism of action and their potential utility in treating atopic dermatitis.

## Materials and methods

### Establishment of MC903-induced atopic dermatitis in C57/BL6 mice

Female C57/BL6 mice, aged six to 8 weeks, were used for all experiments. The mice were housed in an animal barrier system with a temperature of 22°C ± 1°C, a humidity of 55% ± 5%, and a 12-h light and dark cycle. After 1 week of acclimatization, the mice were used for the experiments. All animal experiments were conducted in accordance with regulations and approved by the Animal Ethics Committee of China Pharmaceutical University (2021–11–019). Female C57/BL6 mice, weighing an average of 18–20 g, were randomly divided into four groups: control group, model group, positive control group treated with levocetirizine dihydrochloride (LD), and treatment group receiving decoction of qualified Schizonepeta tenuifolia Briq and Saposhnikovia divaricata (Turcz.) Schischk. The mice were housed in a clean animal room. Each mouse in the experiment was topically applied with 10 μL of MC903 (0.1 nmol/μL) on the right ear once a day for 14 days. The blank group was topically applied with an equal amount of anhydrous ethanol, which was utilized in the preparation of the MC903 solution. The control group was i.g. 0.3 mL of physiological saline by gavage, and the treated groups were given 0.3 mL of drug solution at the corresponding concentration for 12 consecutive days. Percutaneous water loss instrument was used to measure the trans epidermal water loss in the ear lesions of mice every 2 days.

## Drugs and reagents

Schizonepeta tenuifolia Briq and Saposhnikovia divaricata (Turcz.) Schischk were purchased from Jiangsu Provincial Hospital of Traditional Chinese Medicine. High-21.6 g, medium-10.8 g and low-5.4 g of each traditional Chinese medicine botanical drug were weighed and be prepared for decoction by a two-step boiling procedure. First Decoction: Boiling each traditional Chinese medicine botanical drug in 160 mL of sterile boiling water for 36 min, yielding 37 mL of decoction. Second Decoction: Adding 73 mL of sterile water to the residue and boiling for an additional 30 min, yielding 12 mL of decoction. Subsequently, the filtrates from the first and second decoctions were mixed and used for oral administration in mice experiments. The calculation formula is shown in Table 1 below.

**TABLE 1:** The specific formula for calculating the drug dose.

JF doses for final dosing	0.06 g/10 g (mice weight)	0.03 g/10 g (mice weight)	0.0015 g/10 g (mice weight)
JF doses for water extraction	21.6 g	10.8 g	5.4 g
The volume of final mixed decoction	49 mL	49 mL	49 mL
Final dosing volume	0.1363 mL/10 g (mice weight)	0.1363 mL/10 g (mice weight)	0.1363 mL/10 g (mice weight)
Calculation formula	$\text{JF doses for final dosing}=\frac{\text{JF}\;\text{doses}\;\text{for}\;\text{water}\;\text{extraction}\;\left(g\right)}{\text{The}\;\text{volume}\;\text{of}\;\text{final}\;\text{mixed}\;\text{decoction}\;\left(\text{mL}\right)}*\text{Final dosing volume}$ e.g., 0.06 g = (21.6 g/49 mL) *0.136 mL		

For cell experiment, we prepared the powder of both water extraction and ethanol extraction. For ethanol extract of each herb, 20 g of each botanical drug powder was extracted separately by refluxing with 10 times the volume of 70% ethanol for 2 h, for a total of three extractions. The combined extract was filtered and concentrated to a thick paste under reduced pressure (45°C, 60 r·min−1), and then freeze-dried to obtain Schizonepeta tenuifolia Briq and Saposhnikovia divaricata (Turcz.) Schischk alcohol extract. A water extract of Schizonepeta tenuifolia Briq and Saposhnikovia divaricata (Turcz.) Schischk was also prepared using a similar method (Figure 4A). The calculation formula is presented as below:

$$In\;vitro\text{ experimental dosage}=\frac{\text{Lyophilized}\;\text{extract}\;\text{powder}\;\left(g\right)}{\text{Solvent}\left(\text{Appropriate}\;\text{volume}\;\text{of}\;\text{PBS}\right)\left(\text{mL}\right)}$$

For *in vitro* experiment, a certain amount of the freeze-dried powder was dissolved in PBS to a suitable concentration. MC903 (HY-10001, NJ, United States) and levocetirizine dihydrochloride (LD) (HY-W010841, NJ, United States) were purchased from MCE (MedChemExpress) The Schizonepeta tenuifolia Briq and Saposhnikovia divaricata (Turcz.) Schischk medicine has been. The plasmid pCMV-EGFP-TRPV1(mouse)-3×FLAG-Neo was purchased from Miaoling company in Nanjing for the cell transfection. Western blotting antibodies TRPV1 (#66983-1-Ig), JAK1 (#66466-1-Ig), and STAT6 (#51073-1-AP) were purchased from Proteintech. NF-κB P65 (#sc8008), p-NF-κB P65 (#sc-136548), and GAPDH (#sc-32233) antibodies were purchased from Santa Cruz Biotechnology (Santa Cruz, CA, United States). Trizol lysate and ChamQ SYBR qPCR master mix were purchased from Vazyme Biotech (Nanjing, China).

## Human samples

Skin specimens were obtained from four atopic dermatitis patients (diagnosed with atopic dermatitis) and three healthy adults from Department of Dermatology, Affiliated Hospital of Nanjing University of Chinese Medicine. The study protocol to obtain samples from patients and controls was approved by the Ethnic Committee of the Affiliated Hospital of Nanjing University of Chinese Medicine (2022NL-061–02). All experiments were performed in accordance with relevant guidelines and regulations.

### Quality control and determination of Schizonepeta tenuifolia and Saposhnikovia divaricata

The Schizonepeta tenuifolia and Saposhnikovia divaricata have been purchased and identified by pharmacy from Jiangsu Provincial Hospital of Traditional Chinese Medicine. Additionally, we determined the index plant metabolites of Schizonepeta tenuifolia and Saposhnikovia divaricata through HPLC, and confirmed that the main plant metabolites include alkaloids, flavonoids, organic acids, glycosides, and cyclic terpenes. Some of these plant metabolites have been accurately measured for quality and validated according to the existing reference standards in Chinese Pharmacopoeia. In order to prepare adequate samples for HPLC and GC, we first precisely weigh approximately 0.1 g of four types of freeze-dried powder. Specifically, weigh 0.1126 g of ethanol extract of Schizonepeta tenuifolia, 0.0965 g of water extract of Saposhnikovia divaricata, 0.1059 g of water extract of Schizonepeta tenuifolia, and 0.0953 g of ethanol extract of Saposhnikovia divaricata. Place them in a 10 mL volumetric flask, add methanol, ultrasonicate, and then make up the volume to the mark. The concentrations of the sample reference solutions are as follows: Cimifugin: 25 μg/mL; Luteolin: 19.52 μg/mL; Cimigenol: 16.28 μg/mL; Menthone: 10 mg/mL; Pulegone: 10 mg/mL; Octanoic acid: 10 mg/mL; Nonanal: 1.094 mg/mL.

HPLC analysis of indicated samples was performed using Waters ARC HPLC High-Performance Liquid Chromatography system with a 2489 UV/VIS detector. The chromatographic column used is CAPCELL PAK ADME-HR (4.6 mm i.d. × 150 mm) with an injection volume of 5 μL. The column temperature is set at 30°C, and the detection wavelength is 254 nm. The mobile phase consists of A (water) and B (acetonitrile) with a gradient condition for B (acetonitrile) from 0 to 100 min: 5% to 75%.

GC analysis of indicated samples was performed using the Agilent DB-1 System with a dimethylpolysiloxane capillary column (30 m ×length, 250 μm I.D., 0.25 μm thickness). The gas chromatograph is operated at the set temperature: the starting temperature is 100°C, and it is held for 10 min; ramp to 220°C at a rate of 5°C per minute for 10 min; The injector temperature was 250°C, the injection volume was 1 μL, and air, hydrogen, and nitrogen were used as the carrier gases, where the nitrogen flow rate was 25 mL/min, the air flow rate was 400 mL/min, and the hydrogen flow rate was 30 mL/min.

### Hematoxylin and Eosin (HE) staining and toluidine blue staining

After 12 days of treatment, ear tissue samples were taken from each group of mice and fixed in 4% paraformaldehyde solution. The samples were prepared into paraffin sections by embedding, slicing, drying, dewaxing, and hydration, followed by staining with HE or toluidine blue. The tissue structure, inflammatory cell infiltration, and degranulation of dermal mast cells in the skin of each group of mice were observed under an inverted light microscope, and images were collected using a light microscope (OLYMPUS BX53).

### Immunofluorescence staining

The frozen sections were washed in phosphate-buffered saline (PBS), fixed in paraformaldehyde, sealed with sealing solution and subjected to incubation with primary and secondary antibodies followed by staining. Digital composite images were obtained and used to observe the expression of skin barrier-related proteins Involucrin and Filaggrin in the skin of each group of mice under an inverted optical microscope (OLYMPUS IX73). In addition, infiltration of F4/80, CD86, CD206, CD68, and TRPV1 positive cells in the skin of each group of mice was also examined.

### Isolation of primary murine peritoneal macrophages

In order to harvest murine peritoneal macrophages, 6-8-week-old mice were i.p with 1 mL of 4% starch broth into the peritoneal cavity for 4 days. Subsequently, mice were euthanized and injected with PBS into abdominal midline, washed with PBS 2–3 times, repeat this process, centrifuge the obtained exudate to remove the supernatant, and obtain primary peritoneal macrophages.

### Establishment of TRPV1-overexpressing cell line

Transfection was conducted by diluting 2 μg of plasmid pCMV-EGFP-TRPV1(mouse)-3×FLAG-Neo in 100 μL of serum-free medium, and 6 μL of Lipo2000 reagent separately. After incubating at room temperature for 5 min, the two mixtures were thoroughly combined and added to the MEF cells for transfection. The cells were incubated in a 37°C, 5% CO_2_ incubator for 48 h, and the results were validated by Western blotting experiment.

### Establishment of *In vitro* RAW264.7-derived Mφ1 and THP-1-derived Mφ1 cell models

Human myeloid leukemia mononuclear cells (THP-1) were cultured in THP-1 medium containing 15% FBS. The RAW 264.7 in DMEM medium containing 10% FBS. All cells were cultured at 37°C in the 5% CO_2_ atmosphere. Inducing differentiation of cells into Mφ1 macrophages by adding LPS (10 ng/mL) and IFN-γ (10 ng/mL) for 24 h.

### RNA extraction and real-time quantitative PCR

Total RNA was extracted from cells or mouse tissues using Trizol, and complementary DNA (cDNA) was prepared from the total RNA. The quantification of messenger RNA (mRNA) expression levels of IL-4, IL-33, IL-1β, TSLP, IL-6, IL-12/23, TNF-ɑ, CD86, CXCL10, and TRPV1 in cells was detected by quantitative PCR (qPCR) using the ChamQ SYBR qPCR master mix. The relative expression levels of GAPDH in each sample were calculated using the 2^−ΔΔCT^ method. The primer sequences for specific genes are listed in Supplementary Table S1.

### Western blotting

The collected cells were lysed using RIPA lysis buffer to extract total protein. The protein concentration was measured using the BCA assay. Subsequently, the protein samples were denatured by heating at 100°C for 10 min. Equal amounts of total proteins were then loaded onto an SDS-PAGE gel. The proteins were transferred onto a membrane and subsequently blocked with a blocking solution. Following this, the membrane was incubated with primary antibodies, followed by secondary antibodies. Finally, the membrane was visualized by exposing it to an X-ray film. The results were normalized based on the expression of glyceraldehyde 3-phosphate dehydrogenase (GAPDH)**.**

### Statistical analysis

The data were analyzed by GraphPad Prism 9.0 (GraphPad Prism V9.1.0; GraphPad Software, La Jolla, CA, United States). Results are expressed as means ± standard deviation for data adhering to a normal distribution, and as medians (with interquartile range) for data exhibiting a non-normal distribution. Statistical significance was determined using a Student’s t-test or paired one-way ANOVA with Geisser-Greenhouse correction followed by a Tukey-test. *p* values are indicated as: ****p < 0.0001, ***p < 0.001, **p < 0.01, and *p < 0.05.

## Results

### Quality control of Schizonepeta tenuifolia Briq and Saposhnikovia divaricata (Turcz.) Schischk

**FIGURE 1:**
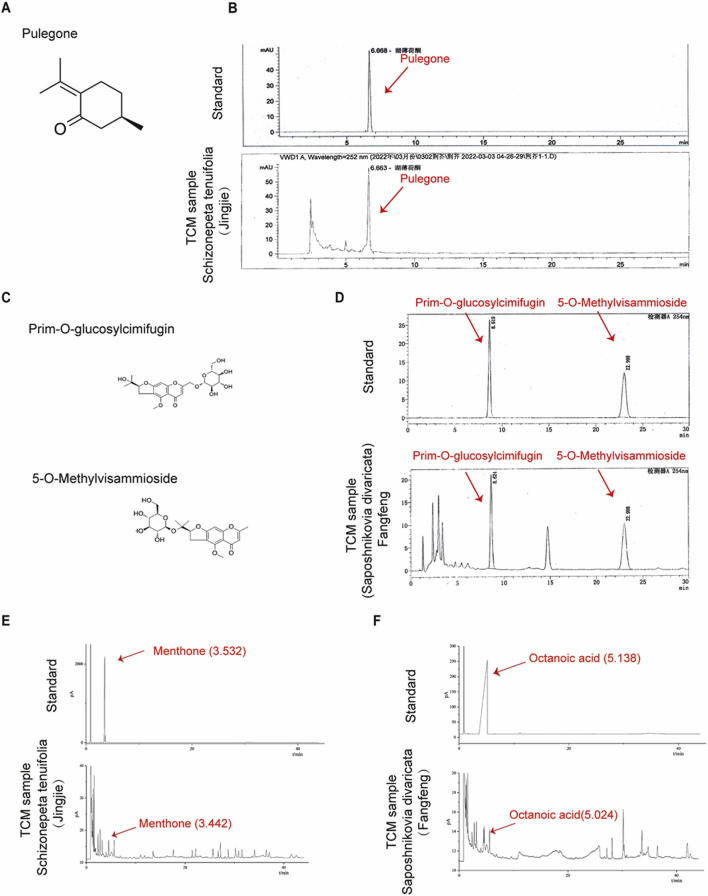
Quantification of Schizonepeta tenuifolia Briq and Saposhnikovia divaricata (Turcz.) Schischk. **(A,B)** Molecular structure of Pulegone and retention time (6.668 min) of Pulegone was determined by HPLC in Schizonepeta tenuifolia Briq. **(C)** Molecular structure of Prim-O-glucosylcimifugin and 5-O-Methylvisammioside. **(D)** Retention time of Prim-O-glucosylcimifugi (8.619 min) and 5-O-Methylvisammioside (22.998 min) were determined by HPLC in Saposhnikovia divaricata (Turcz.) Schischk. **(E)** Water extraction of Schizonepeta tenuifolia Briq was determined by GC. Menthone (3.532 min) was identified as standard. **(F)** Water extraction of Saposhnikovia divaricata (Turcz.) Schischk was determined by GC. Octanoic acid (5.138 min) was identified as standard.

To assess the quality of Schizonepeta tenuifolia Briq (Schizonepeta tenuifolia, Jingjie) and Saposhnikovia divaricata (Turcz.) Schischk (Saposhnikovia divaricata, Fangfeng), we referred to the corresponding indices outlined in the Chinese Pharmacopoeia 2020. Accordingly, we identified Pulegone as the primary active plant metabolites for Schizonepeta tenuifolia and Prim-O-glucosylcimifugin as well as 5-O-Methylvisammioside for Saposhnikovia divaricata (Erdenebileg et al., 2023; Zhao and Zhou, 2022). HPLC analysis revealed that the concentration of Pulegone exceeded the standard concentration specified in the Chinese Pharmacopoeia 2020 (0.104% observed vs. 0.02% standard) (Figures 1A, B). Similarly, the concentration of 5-O-Methylvisammioside and Prim-O-glucosylcimifugin, were found to be higher than the required levels (0.432% observed vs. 0.24% standard, and 0.426% observed vs. 0.24% standard, respectively), as depicted in Figures 1C–F. Furthermore, the concentrations of other plant metabolites within these materials fell below the maximum allowable concentration stipulated in the Chinese Pharmacopoeia 2020 (Table 2). To further verify our water and ethanol extraction, we also run GC analysis on other extraction of Schizonepeta tenuifolia Briq (Jingjie) and Saposhnikovia divaricataSchischk (Fangfeng) (Bai et al., 2021) (Figures 1E–1F). Taken together, our findings indicate that Schizonepeta tenuifolia (Jingjie) and Saposhnikovia divaricate (Fangfeng) meet the requirements outlined in the Chinese Pharmacopoeia 2020, ensuring the reproducibility and stability necessary for subsequent experiments.

**TABLE 2:** Plant ingredients from Schizonepeta tenuifolia Briq and Saposhnikovia divaricata (Turcz.) *Schischk* by available reference standard.

Latin name	English name	Chinese	Reference substance	Standard (%)	Sample (%)
Schizonepeta tenuifolia	Schizonepetae Herba	Jingjie	Pulegone	>0.020	0.1044
Saposhnikovia divaricata	Saposhnikovia divaricata	Fangfeng	Prim-O-glucosylcimifugin 5-O-Methylvisammioside	>0.24	0.858

### JF decoction suppressed MC903-induced atopic dermatotic-like symptoms in mice

**FIGURE 2:**
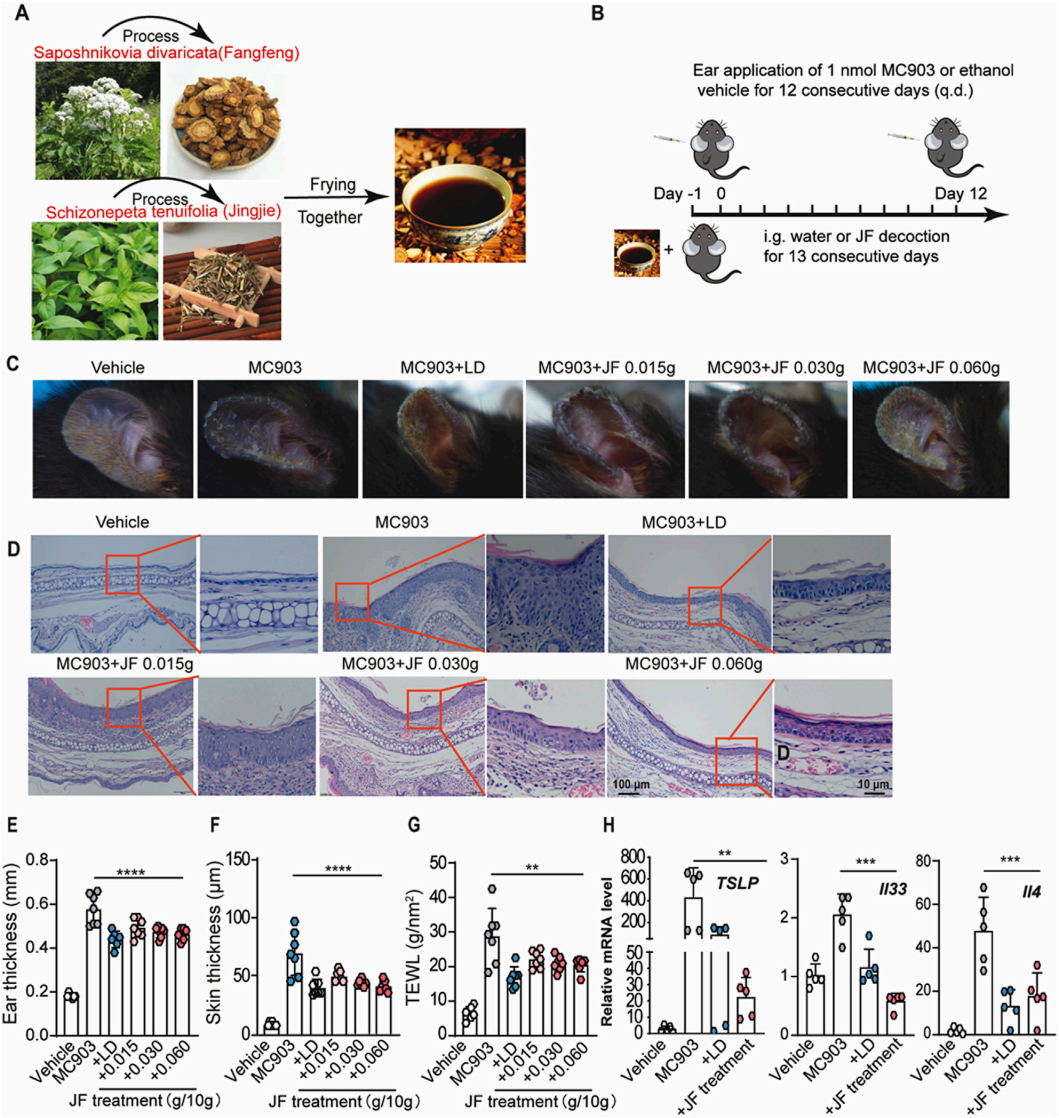
JF decoction suppresses MC903-induced Atopic dermatotic-like symptoms in mice. **(A,B)** Schematic illustration of the process of Schizonepeta tenuifolia -Saposhnikovia divaricata decoction (JF) and application of JF decoction in MC903-induced Atopic dermatotic mice model n = 7. Vehicle control as solution and levocetirizine dihydrochloride (LD) as positive control. **(C)** Representative pictures of ear from MC903-induced atopic dermatitis mice model upon indicated treatment. **(D)** Representative histological pictures of mice ear from various groups (Vehicle, MC903, MC903+LD, MC903+JF). Scale bar = 100 μm and 10 μm. **(E)** Quantification of ear thickness from **(D)** n = 7. **(F,G)** The determination of ear thickness and TEWL were determined in mice upon indicated treatment on day 12 n = 7. **(H)** Quantification of mRNA level of pro-inflammatory cytokines (Tslp, Il33, and Il4) isolated from ear sections of different mice groups (Vehicle, MC903, MC903+LD, MC903+JF). All statistical data are presented as SEM. (n = 5). Statistical significance was calculated by Student’s t-test or one-way analysis of variance (ANOVA) with Tukey’s *post hoc* test. *p < 0.05, **p < 0.01, ***p < 0.001, and ****p < 0.0001, ns: no significance. v.s.MC903 treated group.

To assess the therapeutic efficacy of Schizonepeta tenuifolia - Saposhnikovia divaricata (decoction (hereinafter called JF decoction) intreating atopic dermatitis-like symptoms in a mouse model, we prepared the decoction as described and established the model over a 12-day period (Figures 2A, B). Consistent with clinical observations, the JF decoction significantly alleviated such as scaling and swelling in a dose-dependent manner (Figure 2C). Histological analysis using Hematoxylin and Eosin (HE) staining revealed that JF decoction markedly reduced epidermal thickness and inflammatory cell infiltration in the ears of MC903-induced mice (Figure 2D). To monitor changes in inflammatory status, we measured ear thickness, skin thickness and Transepidermal Water Loss (TEWL) every other day until sacrifice on Day 12. The results showed that the JF decoction effectively reduced ear and skin thickness, as well as TEWL, demonstrating a level of efficacy comparable to that of the positive control, Levocetirizine dihydrochloride (LD) (Figures 2E–G). Further analysis involved evaluating mRNA levels of key atopic dermatitis cytokines in ear tissues. Both the JF decoction and the positive control LD significantly decreased the expression of Tslp, Il33, and Il4, which indicates a suppression of pro-inflammatory cytokine production (Figure 2H). These findings collectively suggest that the JF decoction is an effective treatment for alleviating symptoms similar to those of atopic dermatitis in this mouse model.

### JF decoction recovered the skin barrier impaired in MC903-induced mice model

**FIGURE 3:**
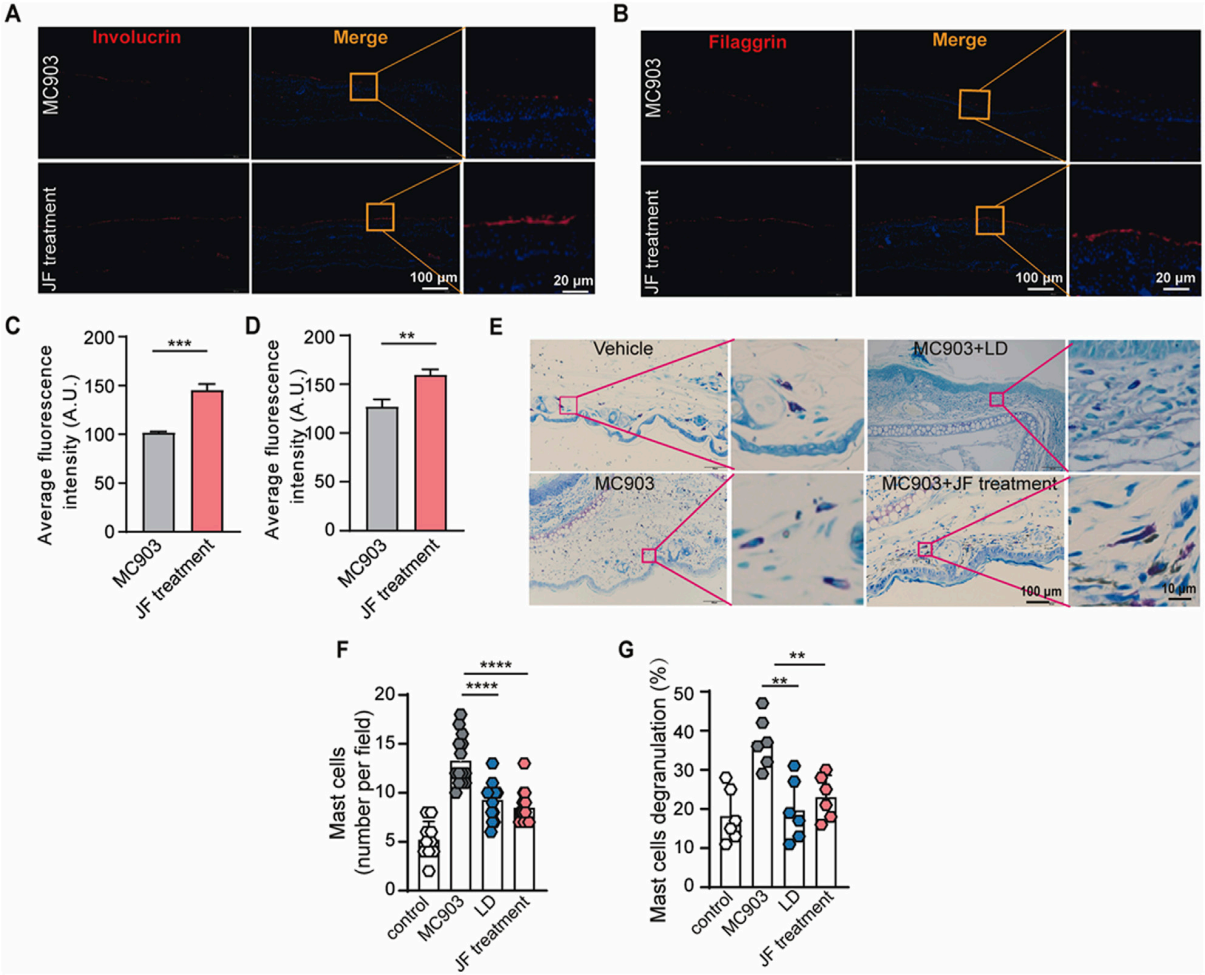
JF decoction recovers the skin barrier damaged by MC903 in atopic dermatitis mouse model. **(A,B)** Representative images of immunofluorescence staining of Involucrin and Filaggrin in mice ear tissue upon indicated treatment (JF, 0.03 g/10 g). Scale bar = 100 μm and 20 μm. **(C,D)** Quantification of Involucrin and Filaggrin in mice ear tissue upon indicated treatment (JF, 0.03 g/10 g) (n = 5). Scale bar-100 μm and 20 μm. **(E–G)** Representative images of Toluidine blue staining and quantification of mast cells in mice ear tissue upon indicated treatment (LD 0.00578 mg/g and JF, 0.03 g/10 g) n = 6 with two PVF counts per individual. n refers to the number of individuals. Scale bar = 100 μm and 10 μm. All statistical data are presented as SEM. Statistical significance was calculated by Student’s t-test or one-way analysis of variance (ANOVA) with Tukey’s *post hoc* test. *p < 0.05, **p < 0.01, ***p < 0.001, and ****p < 0.0001, ns: no significance. v.s.MC903 treated group.

As atopic dermatitis develops, the skin barrier is compromised, leading to physiological changes such as the loss of key barrier proteins, including involucrin (Furue, 2020) and filaggrin (Margolis, 2022), and the activation of mast cells. To investigate the effects of JF treatment on these aspects, we first assessed the integrity of involucrin and filaggrin in the lesional skin of the ears with or without JF treatment. Consistently, our findings demonstrated that JF decoction significantly restored the integrity of involucrin and filaggrin in the skin of MC903-induced mice (Figures 3A–D). Additionally, JF decoction effectively inhibited the infiltration and degranulation of mast cells in the mice (Figures 3E–G). These findings collectively suggest that JF decoction contributes to the restoration of the skin barrier by repairing the damaged epidermis and suppressing mast cell activity.

### JF decoction suppressed macrophage infiltration in MC903-induced atopic dermatitis mouse model

**FIGURE 4:**
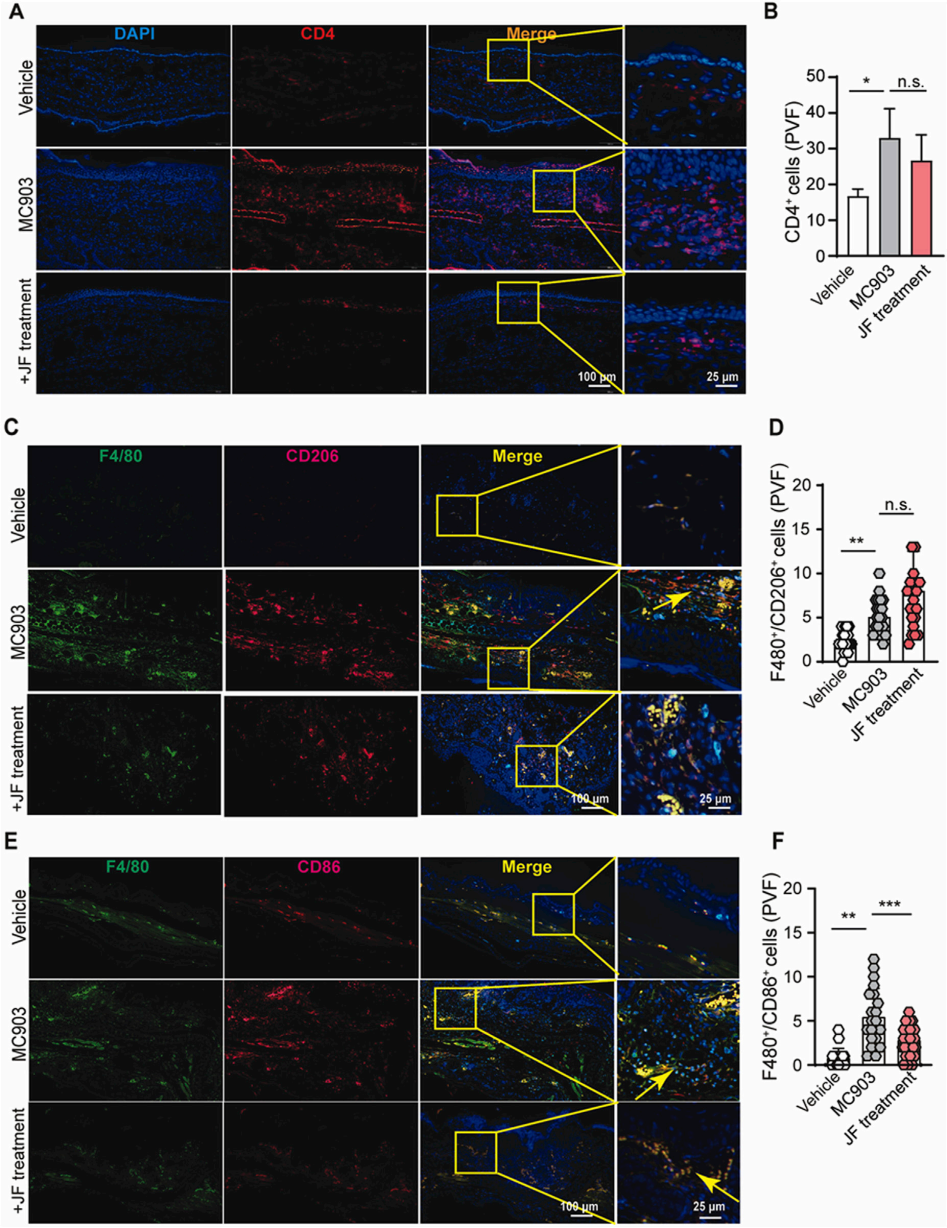
JF decoction suppresses the macrophages infiltration in MC903-induced atopic dermatitis mouse model. **(A,B)** Immunofluorescent staining and quantification of CD4^+^cells in mice skin tissue upon indicated treatment (JF, 0.03 g/10 g). Scale bar = 100 μm and 25 μm. Statistical data were presented as mean ± SEM (n = 5). **(C,D)** Immunofluorescent staining and quantification of F4/80^+^/CD206^+^ cells in mice ear tissue upon indicated treatment (JF, 0.03 g/10 g) (n = 5). Scale bar = 100 μm and 25 μm. **(E,F)** Immunofluorescent staining and quantification of F4/80^+^/CD86^+^ cells in mice ear tissue upon indicated treatment (JF, 0.03 g/10 g) (n = 5). Scale bar = 100 μm and 25 μm n = 5 with three to four PVF counts per individual. n refers to the number of individuals. All statistical data are presented as SEM. Statistical significance was calculated by Student’s t-test. *p < 0.05, **p < 0.01, and ***p < 0.001, ns: no significance. v.s.MC903 treated group.

To explore how the JF decoction affects immune effector cells in atopic dermatitis, we performed staining for dominant immune cells in order to analyze its impact on T cells and macrophages. While the exact pathogenesis of atopic dermatitis remains unclear, it is often considered a type II immunity-related autoimmune disease. Our initial tests focused on T helper (Th) cells, where we noted a modest reduction in the infiltration of CD4^+^ T cells (Figures 4A, B), suggesting that while JF decoction does suppress Th cell infiltration, it may not be the primary mechanism of action in this context.

Understanding the balance between Mφ1 and Mφ2 macrophages in atopic dermatitis is crucial due to their distinct roles in inflammation and tissue repair. Mφ1 macrophages are typically associated with pro-inflammatory responses, exacerbating inflammation and tissue damage in atopic dermatitis. Conversely, Mφ2 macrophages are involved in anti-inflammatory processes and tissue repair, potentially contributing to the resolution of inflammation and restoration of the skin barrier in atopic dermatitis. This balance between Mφ1 and Mφ2 macrophages is essential for understanding both the pathogenesis of atopic dermatitis and the potential effectiveness of treatments. In line with these, our studies showed JF decoction reduced the infiltration of and polarization of macrophages in the atopic dermatitis mice model. Our results indicated that this decoction also reduce the number of infiltrated F4/80^+^/CD86^+^ macrophages (classified as type I macrophages) while slightly increasing the number of infiltrated F4/80^+^/CD206^+^ macrophages (classified as type II macrophages) (Figures 4C–F). Overall, these findings suggest that JF decoction alleviate atopic dermatitis might be more relying on regulating Mφ1-like macrophage in atopic dermatitis progress than Mφ2 macrophage.

### JF water extracts reduced Mφ1-like macrophage activation

**FIGURE 5:**
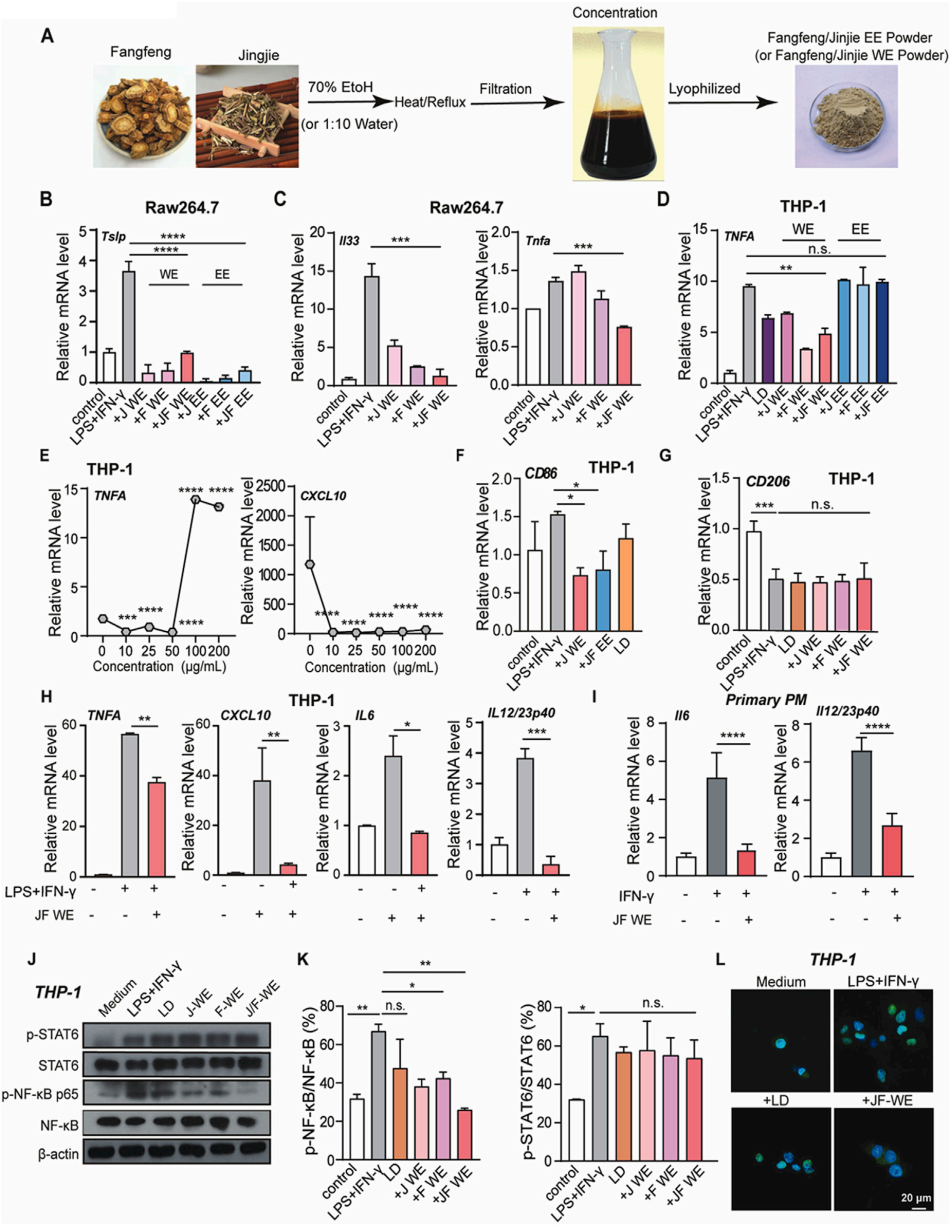
JF extraction suppresses Mγ1 polarization. **(A)** Schematic illustration of the process of JF WE or EE. **(B)** Quantification of mRNA level of atopic dermatitis signature cytokine Tslp in LPS/IFN-γ-induced Raw264.7 cell upon indicated treatment (n = 3) **(C)** Quantification of mRNA level of pro-inflammatory cytokines (Il33 and TNF-ɑ) in LPS/IFN-γ-induced Raw264.7 cell upon indicated treatment. (n = 3) **(D)** Quantification of mRNA level of signature Mφ1 cytokine TNF-ɑ in LPS/IFN-γ-induced THP-1 cells upon indicated treatment. (n = 3) **(E)** Dose-dependent treatment of JF-WE on LPS/IFN-γ-induced THP-1 cells. Quantification of mRNA levels of signature Mφ1 cytokines TNF-ɑ and CXCL10 were determined by q-RT-PCR. (n = 3) **(F)** Quantification of CD86 mRNA expression in THP-1-Mφ1 upon various treatment (JF-WE, 25 μg/mL, JF-EE, 25 μg/mL and LD, 0.0578 μg/mL). (n = 3) **(G)** Quantification of CD206 mRNA expression in THP-1-Mφ1 upon various treatment (J-WE, 25 μg/mL, F-WE, 25 μg/mL, JF-WE, 25 μg/mL and LD, 0.0578 μg/mL). (n = 3) **(H)** THP-1 cells were treated with the indicated dose of JF-WE for 3 h in the presence or absence of LPS and IFN-γ. Measurement of mRNA levels of pro-inflammatory cytokines (CXCL10, IL-6, IL-23, and TNF-ɑ) by q-RT-PCR. (n = 3) **(I)** Primary peritoneal macrophage was isolated, stimulated and then treated with the indicated dose of JF-WE. Measurement of mRNA levels of pro-inflammatory cytokines (IL-6 and IL12/IL-23p40) by q-RT-PCR. (n = 3) **(J,K)** THP-1 cells were pre-treated with indicated therapeutics (LD, 0.0578 μg/mL, JJ-WE, 25 μg/mL, FF-WE 25 μg/mL, JF-WE, 25 μg/mL) with or without stimulation of LPS/IFN-γ. Western blot analysis and quantification of protein expression of p-STAT6/STAT6, p-NF-κB p65/NF-κB p65, and GAPDH. GAPDH was a loading control. (n = 3) **(L)** THP-1 dells were incubated with JF-WE (25 μg/mL) or the same volume of DMSO in the presence of LPS/IFN-γ for 18 h, and then stained with NF-κB p65 (green). The nuclei were stained with DAPI (blue). Scale bar = 20 μm. Data are presented as mean ± SEM; Statistical significance was determined by Student’s t-test or one-way analysis of variance (ANOVA) with Tukey’s *post hoc* test. *p < 0.05, **p < 0.01, ***p < 0.001. n.s., not significant. v.s. LPS + IFN-γ treated group.

To further investigate JF decoction-mediated suppression in macrophages, we prepared two types of extracts using the typical isolation processes of TCM. Ethanol extraction is commonly used to isolate ethanol-soluble plant metabolites such as glycosides, tannins, anthraquinones, and bitter flavors, while water extraction is employed for water-soluble plant metabolites like carbohydrates, glycoside tannins, flavonoid glycosides, and water-soluble plant pigments. Accordingly, we utilized both 70% ethanol and water as reagents to prepare effective extracts from JF decoction (Figure 5A) with quality control (Supplementary Figure S1) and subsequently assessed their efficacy *in vitro*. Our tests revealed that both the individual extracts of Schizonepeta tenuifolia (JJ) and Saposhnikovia divaricate (FF), as well as the combined Schizonepeta tenuifolia - Saposhnikovia divaricate (JF) extracts, effectively suppressed Tslp production in LPS/IFN-γ-stimulated murine macrophage cell line Raw264.7, with minimal differences noted between the ethanol and water extracts (Figure 5B). Given the relevance of these findings to clinical applications, we further investigated whether JF water extract (JF-WE) could suppress LPS/IFN-γ-induced Raw264.7 activation. Indeed, JF-WE significantly suppressed IL33 and TNF-ɑ in LPS/IFN-γ-induced Raw264.7 cells, while single extracts exhibited less pronounced effects (Figure 5C). We extended our investigation to the human macrophage cell line THP-1 and primary peritoneal macrophage (PM), where JF-WE also significantly reduced pro-inflammatory cytokines. Consistent with results from RAW264.7 cells, JF-WE significantly suppressed TNF-ɑ expression in LPS/IFN-γ-induced THP-1 cells (Figure 5D). Furthermore, JF-WE exhibited stronger suppression of Mφ1 macrophage activation within the concentration range of 10 μg/mL to 100 μg/mL, possibly due to the presence of insoluble inclusions in water extracts causing unexpected inflammation at higher doses (Figure 5E). At 50 μg/mL, JF-WE significantly downregulated CD86 expression but had limited impact on CD206 expression in THP-1 cells (Figures 5F, G). Aligned with this, other Mφ1-related pro-inflammatory cytokines (CXCL10, IL-6, TNF-ɑ, and IL-23) at the transcriptional level was decreased by JF-WE treatment in THP-1 cells (Figure 5H). We also verified this observation with primary PM and confirmed that JF-WE treatment can reduce Mφ1-related pro-inflammatory cytokines (Figure 5I).

The JAK-STAT signaling and NF-κB signaling pathways critically involved in the pathogenesis of atopic dermatitis due to their central roles in regulating immune responses and inflammation. The JAK-STAT pathway is involved in the signaling of numerous cytokines implicated in atopic dermatitis, including interleukins (IL-4, IL-13) and interferons (IFNs), which contribute to the pathogenesis of atopic dermatitis by promoting inflammation, epidermal hyperplasia, and barrier dysfunction. Similarly, NF-κB signaling plays a pivotal role in orchestrating the inflammatory response in atopic dermatitis. Activation of NF-κB leads to the production of pro-inflammatory cytokines, chemokines, and adhesion molecules, contributing to the recruitment and activation of immune cells, amplifying inflammation, and perpetuating the pathological processes in atopic dermatitis. Hence, we wondered our JF decoction might downregulate NF-κB signaling pathway or JAK-STAT signaling in macrophage to alleviate atopic dermatitis symptoms and restore skin homeostasis. In this respect, we investigated whether JF-WE regulated inflammatory signaling pathways involved in atopic dermatitis, such as NF-κB and JAK-STAT pathways. In fact, we found JF-WE significantly suppressed NF-κB p65 activation but had a lesser impact on JAK-STAT signaling (Figures 5J, K). Immunofluorescent staining of NF-κB p65 in THP-1 cells further confirmed that JF-WE partially suppressed NF-κB nucleus translocation induced by LPS/IFN-γ stimulation (Figure 5L). Overall, JF water extracts suppresses type I macrophage activation via suppression of NF-κB signaling.

### JF water extracts reduces Mφ1 polarization via downregulation of TRPV1

**FIGURE 6:**
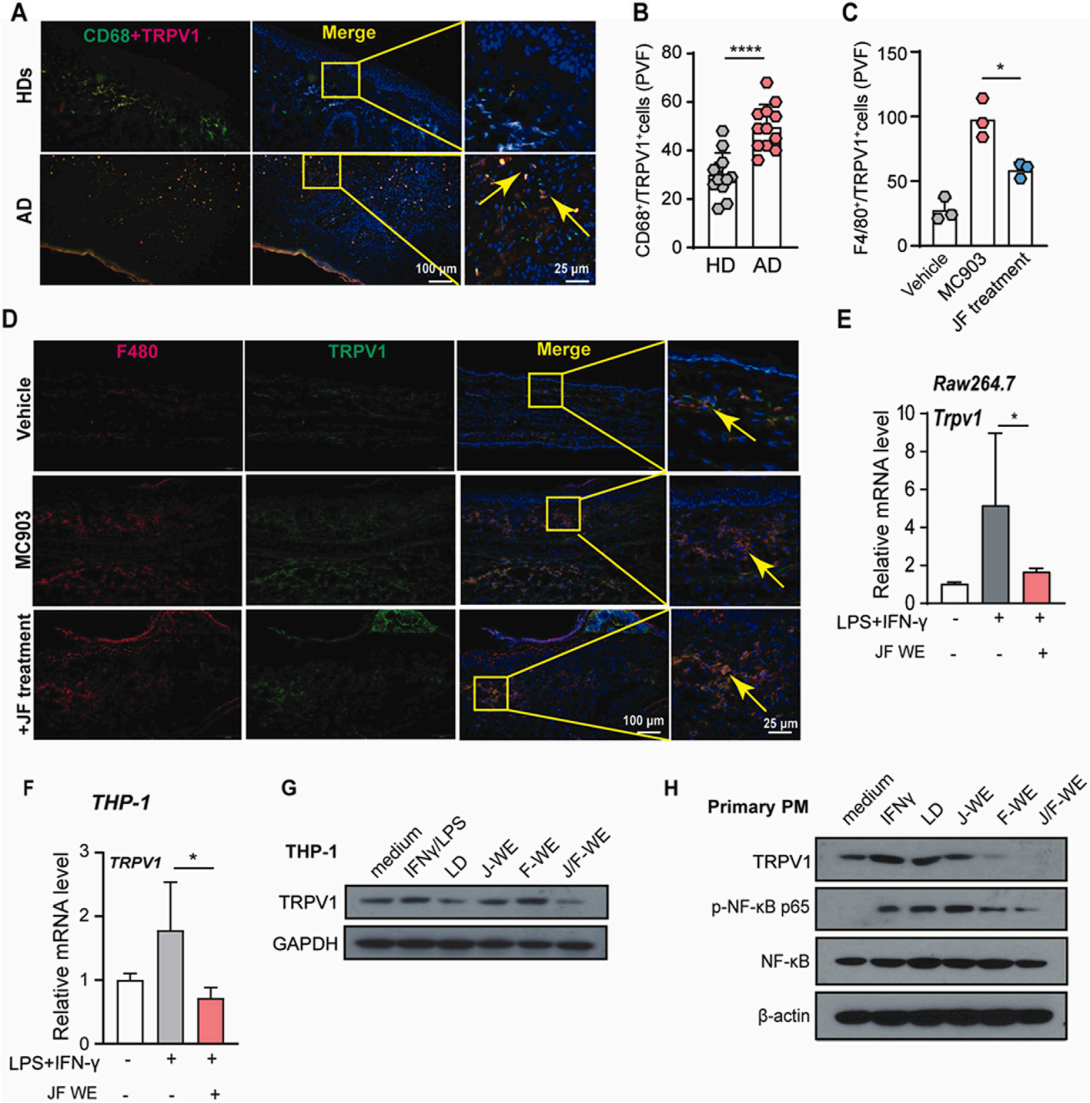
JF water extraction downregulates TRPV1 expression upregulated in Mφ1. **(A,B)** Immunofluorescent staining and quantification of TRPV1^+^/CD68^+^ cells in human atopic dermatitis tissue n = 5, and two PVF count for one individual. **(C,D)** Immunofluorescent staining and quantification of F4/80^+^/TRPV1^+^ cells in mice ear tissue upon indicated treatment (JF, 0.03 g/10 g). (n = 3) Scale bar = 100 μm and 25 μm **(E)** Raw264.7 cells were treated with LPS/IFN-γ in the presence or absence of JF-WE. Quantification of mRNA levels of TRPV1 were determined by q-RT-PCR. (n = 3) **(F)** THP-1 cells were treated with LPS/IFN-γ in the presence or absence of JF-WE. Quantification of mRNA levels of TRPV1 were determined by q-RT-PCR. (n = 3) **(G)** THP-1 cells were pre-treated with indicated therapeutics (LD, 0.0578 μg/mL JJ-WE, 25 μg/mL, FF-WE, 25 μg/mL, JF-WE, 25 μg/mL) with or without stimulation of LPS/IFN-γ. Q-RT-PCR analysis of mRNA expression of TRPV1. Western blot analysis of protein expression of TRPV1 and GAPDH. GAPDH was a loading control. **(H)** Primary peritoneal macrophages were treated with indicated therapeutics (LD, 0.0578 μg/mL JJ-WE, 25 μg/mL, FF-WE, 25 μg/mL, JF-WE, 25 μg/mL) with or without stimulation of IFN-γ. Western blot analysis of protein expression of TRPV1, NF-κB p65, p-NF-κB p65, and β-actin. β-actin was a loading control. Statistical data were presented as mean ± SEM. Statistical significance was calculated by Student’s t-test or one-way analysis of variance (ANOVA) with Tukey’s *post hoc* test. *p < 0.05, **p < 0.01, ***p < 0.001. n.s., not significant. v.s. medium group.

Atopic dermatitis is recognized as an autoimmune disease characterized by dry, itchy, and inflamed skin. The activation of capsaicin receptor TRPV1 has been reported that it is usually associated with itch sensitization as well as well regulating immune cell activity, especially proinflammatory phenotype polarization of macrophages. To investigate this association, we initially examined whether TRPV1 expression was altered in lesional skin tissue from atopic dermatitis patients compared to healthy donors. Indeed, TRPV1 expression was found to be significantly upregulated in skin tissue from atopic dermatitis patients relative to HDs (Figures 6A, B).

Building on this, we investigated the impact of JF water extract (JF-WE) on TRPV1 expression in Mφ1 macrophages within a murine model of atopic dermatitis induced by MC903. The results showed that JF-WE effectively downregulated TRPV1 expression in Mφ1 macrophages in skin tissue from MC903-induced mice (Figures 6C, D). Furthermore, JF-WE significantly suppressed TRPV1 expression in Raw264.7-Mφ1 and THP-1-Mφ1 cells (Figures 6E, F). Comparatively, JF-WE exhibited the strongest suppression of TRPV1 expression compared to other JF extracts and the positive control in both THP-1 cell and primary PM with downregulation of NF-κB p65 phosphorylation (Figures 6G, H). Together, these data suggested that JF water extracts suppresses Mφ1 polarization via downregulation of TRPV1.

### JF water extracts lessened NF-κB p65 phosphorylation via downregulating TRPV1

**FIGURE 7:**
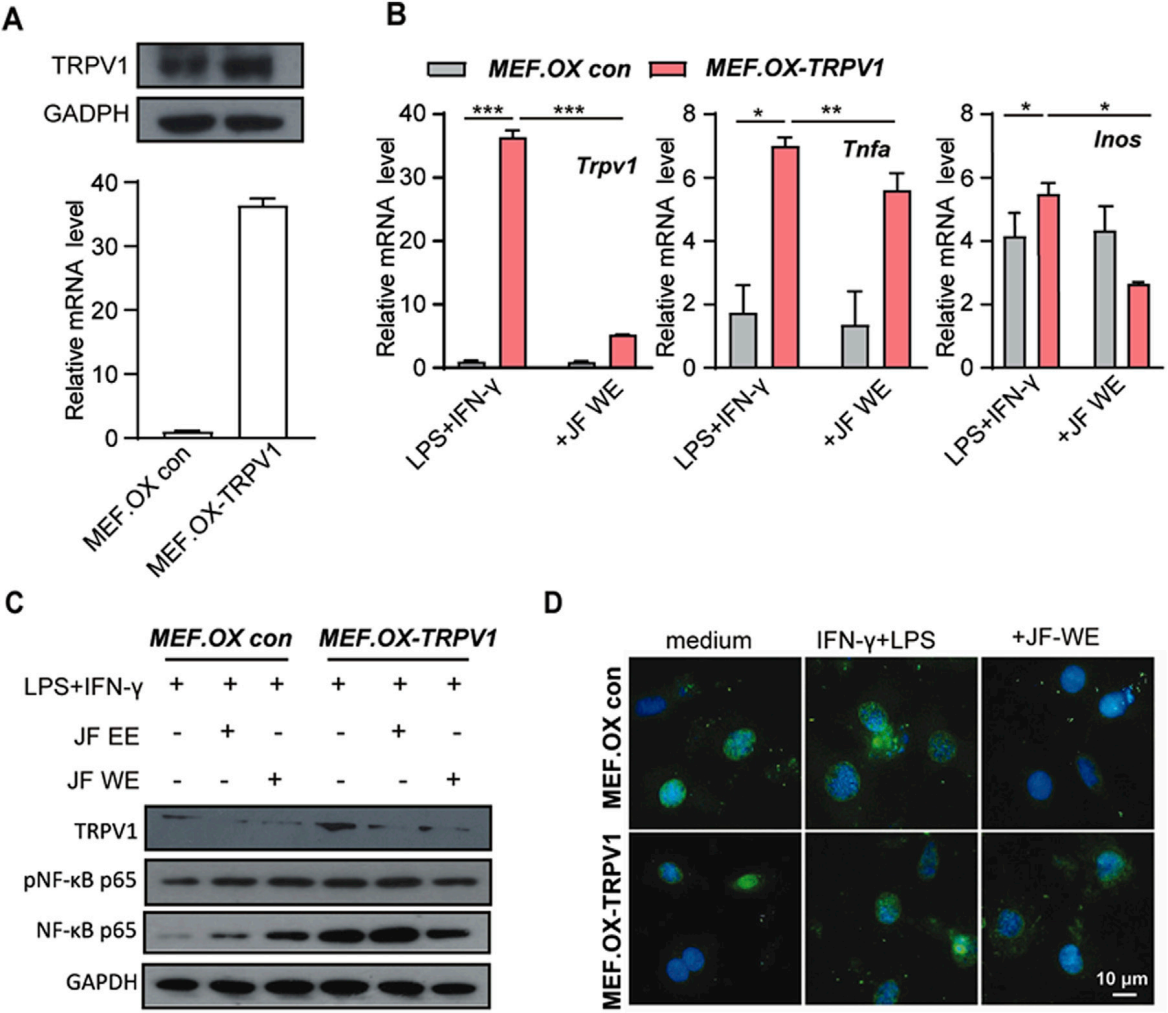
JF water extraction suppresses Mφ1 polarization via downregulation TRPV1. **(A)** The determination of TRPV1-overexpressing MEF cell (MEF.OX TRPV1) and its control MEF.OX con. **(B)** MEF.OX TRPV1 and control cells were pre-treated with JF-WE in the presence or absence of LPS/IFN-γ. Q-RT-PCR of TRPV1, TNF-ɑ, and INOS (n = 3). **(C)** MEF.OX TRPV1 and control cells were pre-treated with indicated therapeutics (JF-EE, 25 μg/mL and JF-WE, 25 μg/mL) with or without stimulation of LPS/IFN-γ. Western blot analysis of protein expression of p-NF-κB p65, NF-κB p65 and GAPDH. GAPDH was a loading control. **(D)** MEF.OX and MEF.TRPV1-OX cells were incubated with JF-WE (25 μg/mL) or the same volume of PBS in the presence of LPS/IFN-γ for 18 h, and then stained with NF-κB p65 (green). The nuclei were stained with DAPI (blue). Scale bar = 10 μm. Statistical significance was calculated by Student’s t-test. *p < 0.05, **p < 0.01, ***p < 0.001. n.s., not significant. v.s. medium group.

To investigate whether JF-WE suppress Mφ1 polarization via downregulation of TRPV1- NF-κB signaling, we first established TRPV1-overexpressing MEF cell line (MEF.TRPV1 ox) and its control cell line (Figure 7A). Previous results showed that increased TRPV1 expression was observed in atopic dermatitis patients compared to HDs and JF-WE treatment inhibited Mφ1 polarization by preventing NF-κB p65 nucleus translocation. To further elucidate this mechanism, we evaluated the effects of JF-WE treatment on TRPV1 and signature Mφ1 markers in LPS/IFN-γ-treated MEF cell pair. Remarkably, JF-WE treatment significantly downregulated TRPV1 expression along with key pro-inflammatory cytokine markers (CXCL10, TNF-ɑ) (Figure 7B). Consistently, JF-WE suppressed NF-κB p65 activation while concurrently downregulating TRPV1 expression (Figure 7C). Immunofluorescence staining of NF-κB p65 in the MEF cell pair revealed that JF-WE partially inhibited NF-κB nucleus translocation induced by LPS/IFN-γ stimulation (Figure 7D). Overall, the suppression of LPS/IFN-γ-induced NF-κB p65 activation by JF-WE may be attributed to the downregulation of TRPV1 expression.

## Discussion

Atopic dermatitis is a complicated immunological disorder depending on the interaction between immune cells and other responding cells. Despite the fact that many western therapeutics have been developed for atopic dermatitis treatment, the prevalence of atopic dermatitis is still increasing (Nutten, 2015). For instance, 15%–20% of children and 10% of adults worldwide suffer from atopic dermatitis, making atopic dermatitis the dermatosis with the highest disease burden, measured in disability-adjusted life-years (Laughter et al., 2021). On one hand, the pathophysiology of atopic dermatitis still remains unclear and inexplicit, which involves the dysfunctional immune cells and epidermal barrier damage (Patrick et al., 2021). On the other hand, Chinese herbal medicine can provide significant improvement in atopic dermatitis symptoms and has been reported to be well tolerated (Tan et al., 2013). Clinically, Xiao-Feng-San has been administered to atopic dermatitis patients as an alternative treatment in many Traditional Chinese Medicine hospitals, yielding promising results (Chen et al., 2015; Cheng et al., 2011; Wang et al., 2021). Xiao-Feng-San can alleviate both the clinical lesion score and pruritus score in refractory atopic dermatitis patients. As the core botanical drugs of traditional Chinese medicine formula Xiao-Feng-San, Schizonepeta tenuifolia Briq and Saposhnikovia divaricata (Turcz.) Schischk are often used as herbal pairs in clinical practice. Our study focused on the therapeutic effects of the JF decoction, a component of Xiao-Feng-San, on an MC903-induced atopic dermatitis -like mice model. The results confirmed that the JF decoction significantly alleviated cutaneous inflammatory symptoms.

Cutaneous inflammation is the essential pathological signature of atopic dermatitis. Meanwhile, atopic dermatitis is also classified as a type II immune disorder, resulting in its increased infiltrated immune effector cells (e.g., Langerhans, ILCs, macrophages, and Th2 cells) and pro-inflammatory cytokines secretion (e.g., IL-4, IL-13, IFN-γ). Amongst all immune effector cells, macrophages have become increasingly important in atopic dermatitis progress (Hashimoto et al., 2023; Rojahn et al., 2020). Although macrophages in reality should be a mixed subgroups with multiple phenotypes, *in vitro* activation of macrophages normally classified into two types, LPS/IFN-γ-stimulated Mφ1 macrophage and IL-4/IL-13-stimulated Mφ2 macrophage. Mφ1 macrophages display pro-inflammatory features and activate/attract T cells, whereas Mφ2 macrophages display the opposite features which are regarded as anti-inflammatory macrophages. Our results suggest JF decoction significantly suppressed infiltrated Mφ1 macrophages and slightly increased infiltrated Mφ2 macrophages in lesioned skin from atopic dermatitis -like mice model (Figure 3). This intriguing phenomenon indicated JF decoction might not directly suppress Mφ2-Th2 axis like other biological reagents but tend to reduce the inflammatory atopic dermatitis skin microenvironment. Accordingly, instead of focusing on JF-effect on Mφ2-like macrophage, we further shifted our sight towards whether JF modulated macrophage activation and corresponding pathways. Indeed, JF extract notably inhibits LPS/IFN-γ-stimulated Mφ1 polarization, suggesting that therapeutics targeting Mφ1 activation may serve as a viable alternative for treating patients with atopic dermatitis.

Previously (Shi et al., 2021), we analyzed the potential signaling pathways behind JF capability in atopic dermatitis treatment by network pharmacology, and the results suggested TNF-ɑ, IL-6 etc., as well as the corresponding predominant pathways (e.g., NF-κB and JAK- STAT6) were the key targets of JF in atopic dermatitis treatment. In line with these, we first explored which pathways in macrophages were more impacted by JF extracts. By evaluating their regulation in phosphorylation of NF-κB and STAT6, we found JF-WE displayed the optimal efficacy and downregulation of NF-κB p65 activity in murine and human macrophages, suggesting this extract might be the best in suppressing Mφ1 polarization via inhibiting NF-κB phosphorylation (Figure 4).

Numerous studies purposed that atopic dermatitis was a type II immune disorder in which IL-4/IL-13 are the most essential cytokines involved in the pathogenesis of disease. However, other potential molecular factors have been proved to contribute to the development of atopic dermatitis. One of the particular and intriguing protein is Transient receptor potential (TRP). TRPs as ion channels are widely distributed in the skin which are involved in modulating skin differentiation and maintaining skin barrier such as TRPV1 (Meng et al., 2021; Tang et al., 2022). TRPV1 activation delayed skin barrier repair via modulation calcium ion concentration. Therefore, it has been considered as a potential target for prevention and treatment of atopic dermatitis. In this respect, some therapeutics have been developed and applied in clinical trial of patients with atopic dermatitis, such as TRPV1 antagonist PAC-14028 evaluated in a phase 2 clinical trial (Lee et al., 2019). In this context, our findings reveal that TRPV1 expression is upregulated in Mφ1 macrophages, and this upregulation can be mitigated by JF water extract. Our findings confirm that JF treatment can downregulate TRPV1 expression in Mφ1 macrophages, suggesting that JF could potentially counteract TRPV1 activation, thereby suppressing Mφ1 macrophage activity and contributing to its therapeutic efficacy in atopic dermatitis.

## Conclusion

Taken together, this study discovered that the herbal pair Schizonepeta tenuifolia Briq-Saposhnikovia divaricata (Turcz.) Schischk (JF) from TCM Xiao-Feng-San-formula could significantly ameliorate the cutaneous inflammatory symptoms and skin lesions of MC903-induced atopic dermatitis -like mice. Furthermore, JF extract suppressed Mφ1 polarization by downregulating TRPV1 expression, consequently inhibiting suppression on NF-κB p65 phosphorylation. These results suggest that JF, as the core combination from Xiao-Feng-San-formula, may offer therapeutic benefits for patients with atopic dermatitis.

## Figures and Tables

**Table:** 

**JF**	Schizonepeta tenuifolia Briq-Saposhnikovia divaricata (Turcz.) Schischk
**MC903**	Calcipotriene
**HPLC**	High-performance liquid chromatography
**GC**	Gas chromatography
**TEWL**	Trans Epidermal Water Loss
**TSLP**	Thymic stromal lymphopoietin
**IL-33**	Interferon-33
**IgE**	Immunoglobulin E
**TRPV1**	Transient Receptor Potential Cation Channel Subfamily V Member 1
**INOS**	Inducible Isoform of Nitric Oxide Synthase
**TNFɑ**	Tumor necrosis factor-α; CXCL10
**CXCL10**	CXCchemokineligand-10
**LPS**	Lipopolysaccharide
**IFN-γ**	Interferon-γ
**WE**	Water extract
**NF-κB**	nuclear factor-κ-gene binding
**IL-22**	interferon-22
**IL-4**	interferon-4
**IL-13**	Interferon-13
**JAK**	Janus kinase
**STAT**	Signal transducer and activator of transcription
**IL-23**	interferon-23
**HDs**	Healthy donors
**TRP**	Transient receptor potential
**EASI 90**	eczema area and severity index reduction of 90%
**LD**	Levocetirizine dihydrochloride
**GAPDH**	Glyceraldehyde-3-phosphate dehydrogenase
**H&E**	Haematoxylin and eosin
**IF**	Immunofluorescence
**IL-1β**	interferon-1β
**IL-6**	interferon-6
**CD86**	Cluster of Differentiation 86
**F4/80**	mouse EGF-like module-containing mucin-like hormone receptor-like 1
**CD206**	Macrophage mannose receptor 1
**TCM**	Traditional Chinese Medicine
**PBS**	Phosphate buffered saline
**RT-PCR**	Reverse transcription polymerase chain reaction
**SDS**	Sodium dodecyl sulfate
**BSA**	Bovine serum albumin
**DAPI**	4′,6-Diamidino-2-phenylindole
**ECL**	Enhanced chemiluminescence
**FBS**	Fatal bovine serum
**Mφ1**	M1-polarized macrophages
**Mφ2**	M2-polarized macrophages

## Data Availability

The original contributions presented in the study are included in the article/Supplementary Material, further inquiries can be directed to the corresponding authors.
