# Supplementary material for: Schizonepeta tenuifolia Briq-Saposhnikovia divaricata decoction alleviates atopic dermatitis via downregulating macrophage TRPV1
Source: Front Pharmacol. 2024 Aug 27;15:1413513. doi: 10.3389/fphar.2024.1413513 (PMC11383762; doi:10.3389/fphar.2024.1413513)
Supplement: Supplementary file 1 [file Table1.docx]

**Supplementary Table S1 Sequences of target gene specific primer**

| Gene name |  | Primer sequences (5'-3') |
| --- | --- | --- |
| Mouse GAPDH  NM_001289726.2 | Forward | 5'-AGGTCGGTGTGAACGGATTTG-3' |
|  | Reverse | 5'-TGTAGACCATGTAGTTGAGGTCA-3' |
| Mouse IL-4  NM_021283.2 | Forward | 5'-GGTCTCAACCCCCAGCTAGT-3' |
|  | Reverse | 5'-GCCGATGATCTCTCTCAAGTGAT-3' |
| Mouse IL-33  NM_001164724.2 | Forward | 5'-TCCAACTCCAAGATTTCCCCG-3' |
|  | Reverse | 5'-CATGCAGTAGACATGGCAGAA-3' |
| Mouse TSLP  NM_021367.2 | Forward | 5'-ATGATTCTCCGACTCATTGCAC-3' |
|  | Reverse | 5'-GAGGATCACGTAATGGGGCTT-3' |
| Mouse TNF-ɑ  NM_001278601.1 | Forward | 5'-GAGTCCGGGCAGGTCTACTTT-3' |
|  | Reverse | 5'-CAGGTCACTGTCCCAGCATCT-3' |
| Mouse TRPV1  NM_001001445.2 | Forward | 5'-CCACTGGTGTTGAGACGCC-3' |
|  | Reverse | 5'-TCTGGGTCTTTGAACTCGCTG-3' |
| Human GAPDH  NM_001256799.3 | Forward | 5'-ACAACTTTGGTATCGTGGAAGG-3' |
|  | Reverse | 5'-GCCATCACGCCACAGTTTC-3' |
| Human IL-6  NM_000600.5 | Forward | 5'-ACTCACCTCTTCAGAACGAATTG-3' |
|  | Reverse | 5'-CCATCTTTGGAAGGTTCAGGTTG-3' |
| Human TNF-ɑ  NM_000594.4 | Forward | 5'-CCTCTCTCTAATCAGCCCTCTG-3' |
|  | Reverse | 5'-GAGGACCTGGGAGTAGATGAG-3' |
| Human CXCL10  NM_001565.4 | Forward | 5'-GTGGCATTCAAGGAGTACCTC-3' |
|  | Reverse | 5'-TGATGGCCTTCGATTCTGGATT-3' |
| Human CD86  NM_001206924.2 | Forward | 5'-CTGCTCATCTATACACGGTTACC-3' |
|  | Reverse | 5'-GGAAACGTCGTACAGTTCTGTG-3' |
| Human IL-12/23  NM_002187.3 | Forward | 5'-CTCAGGGACAACAGTCAGTTC-3' |
|  | Reverse | 5'-ACAGGGCTATCAGGGAGCA-3' |
| Human TRPV1  NM_080706.3 | Forward | 5'-CAGGCTCTATGATCGCAGGAG-3' |
|  | Reverse | 5'-TTTGAACTCGTTGTCTGTGAGG-3' |
