# Supplementary figures and images for: Schizonepeta tenuifolia Briq-Saposhnikovia divaricata decoction alleviates atopic dermatitis via downregulating macrophage TRPV1
Source: Front Pharmacol. 2024 Aug 27;15:1413513. doi: 10.3389/fphar.2024.1413513 (PMC11383762; doi:10.3389/fphar.2024.1413513)

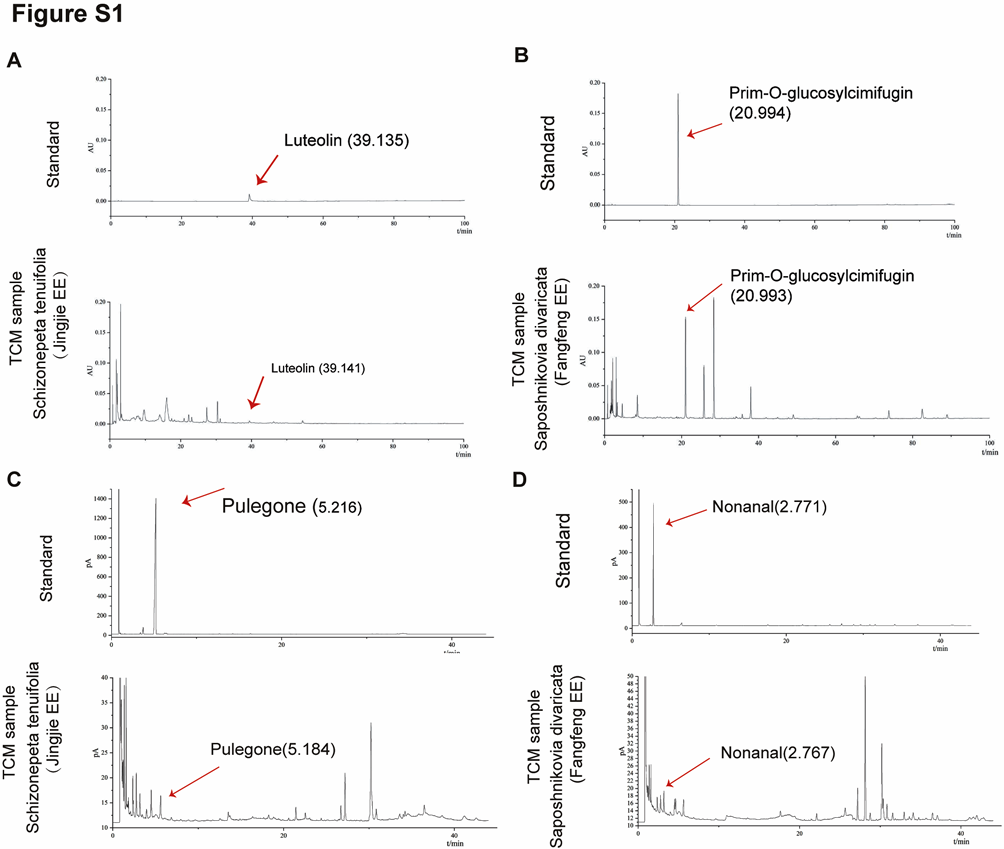

Supplement: Supplementary file 2 [file Image1.tif]
